# Negative association between multiple sclerosis immunogenetic profile and in silico immunogenicities of 12 viruses

**DOI:** 10.1038/s41598-023-45931-5

**Published:** 2023-10-31

**Authors:** Lisa M. James, Apostolos P. Georgopoulos

**Affiliations:** 1 The HLA Research Group, Brain Sciences Center, Department of Veterans Affairs Health Care System, Minneapolis, MN 55417 USA; 2 Department of Neuroscience, University of Minnesota Medical School, Minneapolis, MN 55455 USA; 3 Department of Psychiatry, University of Minnesota Medical School, Minneapolis, MN 55455 USA; 4 Department of Neurology, University of Minnesota Medical School, Minneapolis, MN 55455 USA

**Keywords:** Computational biology and bioinformatics, Immunology, Molecular biology, Neuroscience, Medical research, Neurology

## Abstract

Human Leukocyte Antigen (HLA) is involved in both multiple sclerosis (MS) and immune response to viruses. Here we investigated the virus-HLA immunogenicity (V-HLA) of 12 viruses implicated in MS with respect to 17 HLA Class I alleles positively associated to MS prevalence in 14 European countries. Overall, higher V-HLA immunogenicity was associated with smaller MS-HLA effect, with human herpes virus 3 (HHV3), JC human polyoma virus (JCV), HHV1, HHV4, HHV7, HHV5 showing the strongest association, followed by HHV8, HHV6A, and HHV6B (moderate association), and human endogenous retrovirus (HERV-W), HHV2, and human papilloma virus (HPV) (weakest association). These findings suggest that viruses with proteins of high HLA immunogenicity are eliminated more effectively and, consequently, less likely to be involved in MS.

## Introduction

Multiple sclerosis (MS) is a chronic autoimmune inflammatory disease which affects the central nervous system and is characterized by multifocal demyelinating lesions, axonal loss, and atrophy^1^. MS is the most common neurological disorder among young adults and its global prevalence is increasing for unclear reasons^2,3^. The etiology of MS is uncertain although viruses have long been purported to contribute to the disease, particularly in genetically vulnerable individuals. For example, human herpes viruses (HHV) including Epstein-Barr virus (EBV/HHV4), roseolavirus (HHV6), and varicella zoster virus (VZV/HHV3) as well as human endogenous retroviruses (HERVs) have been commonly implicated in MS^4–7^. In addition, human polyoma JC virus (JCV), a human polyomavirus, is associated with MS and particularly in complications stemming from immunosuppressive treatment for MS^8–10^. The primary genetic influence on MS is attributed to human leukocyte antigen (HLA) genes which are centrally involved in the human immune response to viruses and other foreign antigens and have been implicated in both MS risk and protection^11–16^. In a recent immunogenetic epidemiological study, we evaluated the association between the population frequencies of 127 HLA alleles and the population prevalence of MS across 14 European countries and found a preponderance of negative (i.e., protective) associations between HLA allele frequencies and MS prevalence, particularly for Class I HLA alleles^16^. Given the role of HLA in elimination/suppression of viruses and other foreign antigens, we hypothesized that negative (i.e., protective) associations between Class I HLA and MS are likely attributable to superior pathogen elimination afforded by those alleles, and that, conversely, positive (i.e., susceptibility) HLA-MS associations may be attributable to insufficient immunogenetic protection against certain pathogens, thereby hindering their suppression and possibly contributing to downstream effects associated with MS. Here, in an effort to test this hypothesis and bridge separate lines of research implicating exposure to pathogens and HLA in MS, we evaluated the virus-HLA (V-HLA) immunogenicity of viruses implicated in MS with respect to HLA alleles that are positively associated with MS prevalence.

## Results

### MS-HLA susceptibility scores

**Figure 1:**
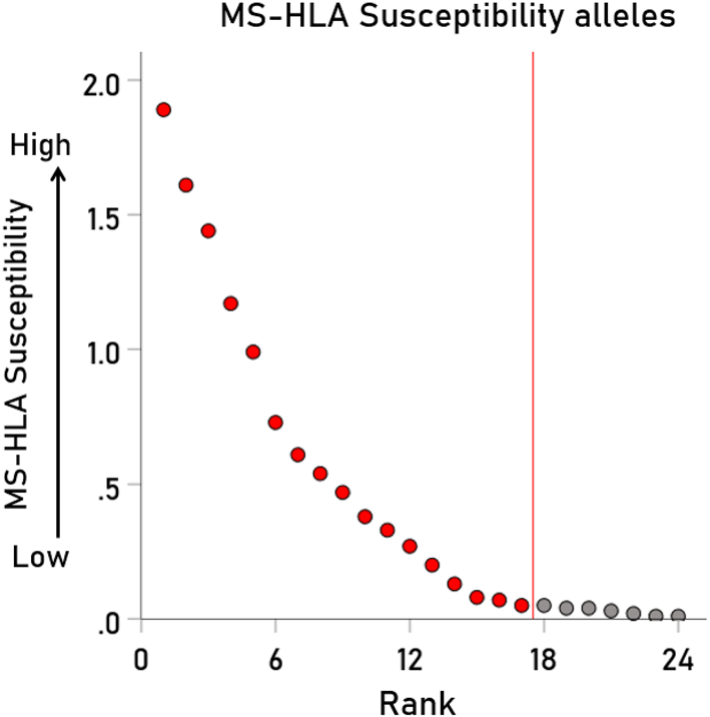
MS-HLA susceptibility scores are plotted against their rank. Red, scores of alleles used I further analyses; gray, scores at the tail of the distribution, not used hereafter. The red line demarcates these two groups. See text for details.

The MS-HLA Susceptibility scores are epidemiological measures of association between MS prevalence and HLA allele frequency. Of the 69 HLA Class I alleles investigated, 24 were positive, indicating a positive association between MS prevalence and allele frequency (Fig. 1). It can be seen that the scores were practically the same for the last 7 alleles and, hence, the scores of the top 17 alleles (Table 1) were used for further analyses.

**Table 1:** MS-HLA PScov scores for the 17 susceptibility Class I alleles investigated.

Index	Allele	MS-HLA PScov score (× 1000)
1	A*03:01	1.890
2	B*07:02	1.606
3	B*15:01	1.435
4	B*27:05	1.171
5	B*40:01	0.993
6	C*07:02	0.726
7	A*24:02	0.612
8	A*31:01	0.538
9	C*05:01	0.465
10	C*03:03	0.380
11	B*40:02	0.331
12	B*37:01	0.273
13	C*16:01	0.204
14	B*08:01	0.127
15	C*01:02	0.079
16	A*25:01	0.066
17	A*68:02	0.051

### Immunogenicity of viral proteins for HLA Class I alleles

**Figure 2:**
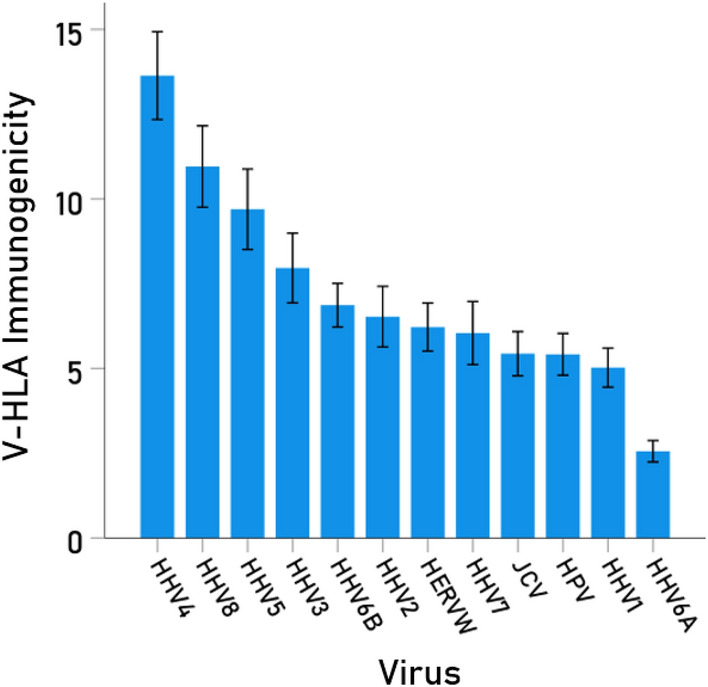
Mean (± SEM) of V-HLA immunogenicity scores for each one of the 12 viruses investigated (N = 17 alleles in Table 1).

**Figure 3:**
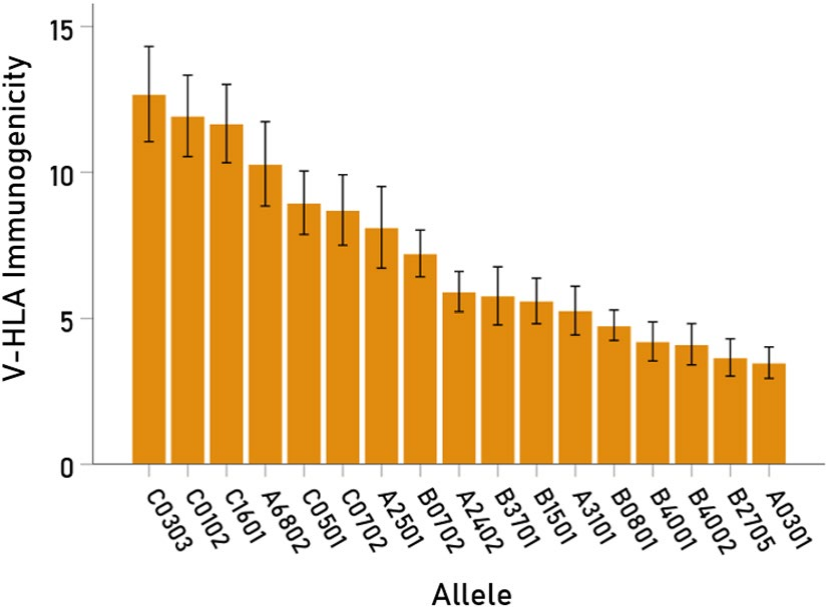
Mean (± SEM) of MS-HLA susceptibility scores for each one of the 17 alleles in Table 1. (N = 12 viruses in Fig. 2).

In silico virus-HLA immunogenicity scores (V-HLA scores) are estimates of T-cell epitope prediction, indicating the likelihood that the complex between a given epitope and a specific HLA Class I allele will engage T-cell receptor and, hence, activate CD8 + cytotoxic lymphocytes to kill the infected cell. V-HLA immunogenicity varied appreciably among the 12 viruses studied (Table 2, Fig. 2), being highest for HHV4 (V-HLA = 13.639) and lowest for HHV6A (V-HLA = 2.563), a 5.32 × differential. V-HLA was highest for allele C*03:03 (V-HLA = 12.686) and lowest for A*03:01 (V-HLA = 3.486) (Table 3, Fig. 3).

**Table 2:** Descriptive statistics of V-HLA immunogenicities across the 17 HLA Class I alleles in Table 1 (N = 17).

Rank	Virus	Mean	SEM	Minimum	Maximum
1	HHV4	13.639	1.295	7.348	24.922
2	HHV8	10.959	1.200	3.886	19.738
3	HHV5	9.697	1.185	3.722	17.744
4	HHV3	7.965	1.026	2.708	18.002
5	HHV6B	6.869	0.644	3.960	13.480
6	HHV2	6.532	0.894	2.612	14.420
7	HERVW	6.223	0.709	1.854	11.350
8	HHV7	6.049	0.931	1.422	12.198
9	JCV	5.439	0.651	1.004	11.228
10	HPV	5.421	0.616	1.590	8.994
11	HHV1	5.027	0.577	1.966	9.484
12	HHV6A	2.563	0.316	0.460	4.632

*SEM* standard error of the mean.

**Table 3:** Descriptive statistics of V-HLA immunogenicities across the 12 viruses investigated for the 17 Class I alleles in Table 1..

Rank	Allele	Mean	SEM	Minimum	Maximum
1	C*03:03	12.686	1.633	3.794	24.922
2	C*01:02	11.937	1.398	4.246	20.882
3	C*16:01	11.674	1.343	3.790	21.294
4	A*68:02	10.290	1.447	3.472	18.002
5	C*05:01	8.957	1.088	3.898	15.304
6	C*07:02	8.706	1.209	3.338	16.462
7	A*25:01	8.112	1.398	3.208	17.744
8	B*07:02	7.219	0.803	2.076	13.208
9	A*24:02	5.911	0.688	2.830	10.240
10	B*37:01	5.771	0.987	1.734	11.688
11	B*15:01	5.595	0.773	2.556	11.482
12	A*31:01	5.267	0.828	0.474	12.432
13	B*08:01	4.763	0.514	2.030	8.116
14	B*40:01	4.217	0.671	1.594	8.540
15	B*40:02	4.120	0.707	1.640	9.272
16	B*27:05	3.665	0.639	1.124	8.746
17	A*03:01	3.486	0.539	0.460	7.348

*SEM* standard error of the mean.

### Association between MS-HLA susceptibility and V-HLA immunogenicity

**Figure 4:**
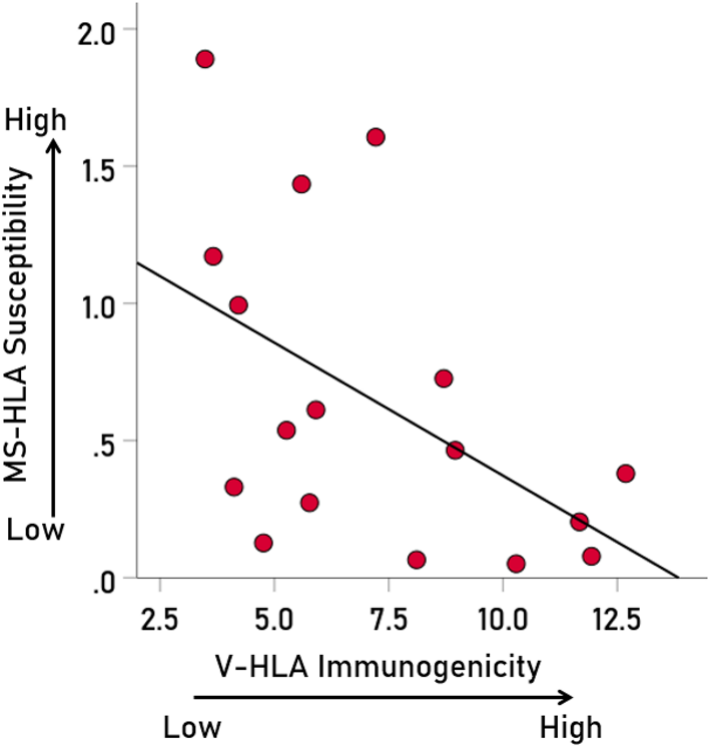
The MS-HLA susceptibility scores of the 17 alleles (Table 1) are plotted against the mean of the corresponding (per allele) V-HLA immunogenicity scores (N = 12 viruses). See text for details.

**Figure 5:**
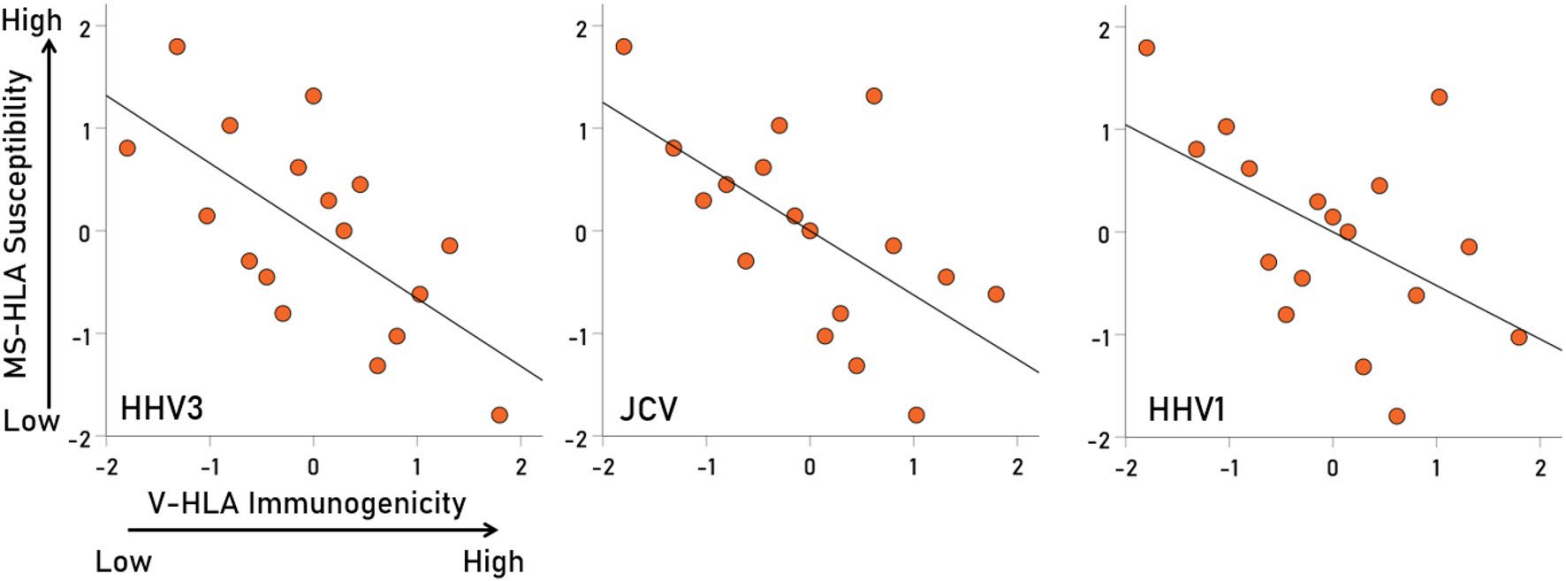
Negative association of MS-HLA susceptibility scores of the 17 alleles (Table 1) vs. corresponding V-HLA immunogenicity scores for the viruses indicated (HHV3, JCV, HHV1). See Table 4 for detailed statistics.

**Figure 6:**
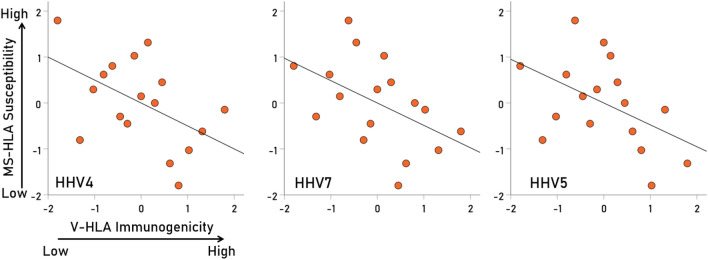
Negative association of MS-HLA susceptibility scores of the 17 alleles (Table 1) vs. corresponding V-HLA immunogenicity scores for the viruses indicated (HHV4, HHV7, HHV5). See Table 4 for detailed statistics.

**Figure 7:**
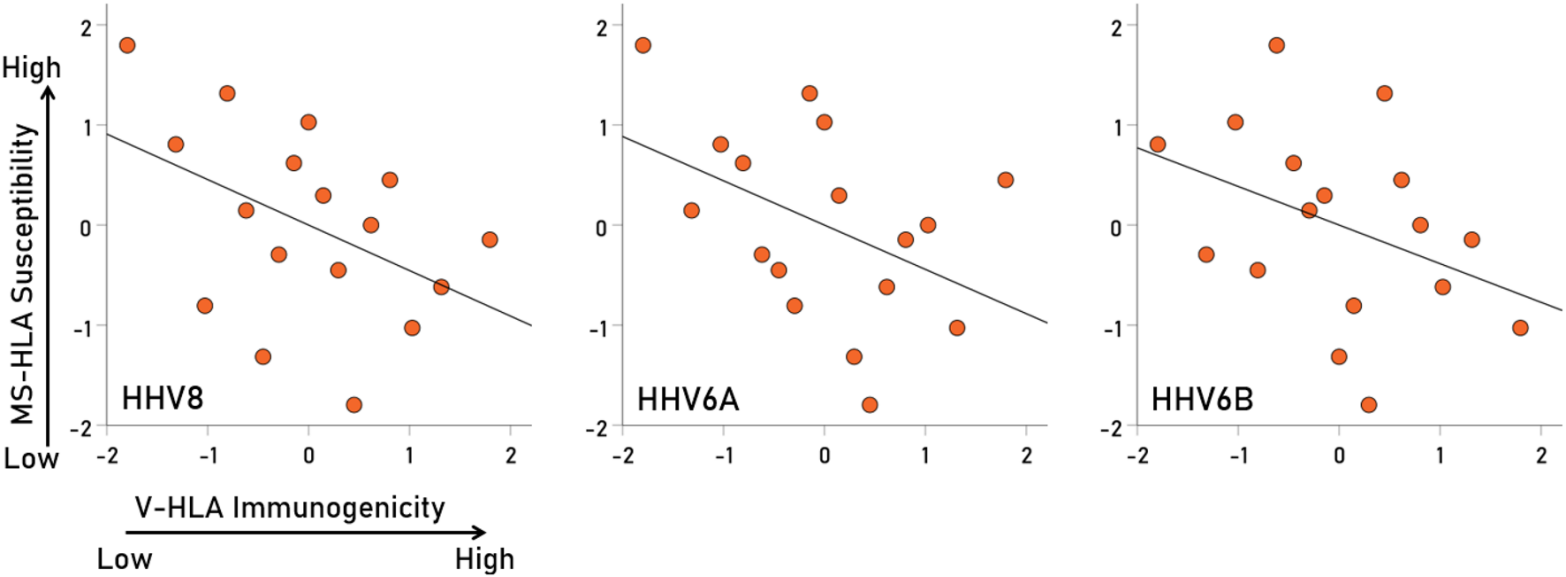
Negative association of MS-HLA susceptibility scores of the 17 alleles (Table 1) vs. corresponding V-HLA immunogenicity scores for the viruses indicated (HHV8, HHV6A, HHV6B). See Table 4 for detailed statistics.

**Figure 8:**
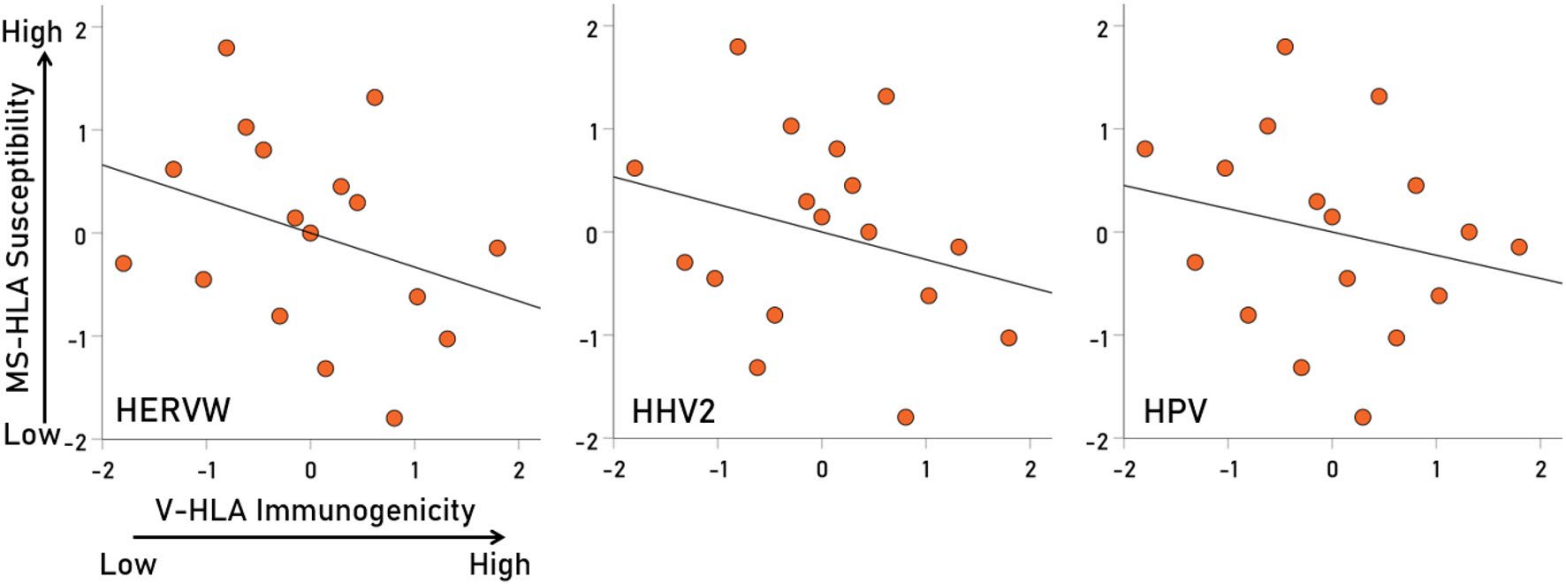
Negative association of MS-HLA susceptibility scores of the 17 alleles (Table 1) vs. corresponding V-HLA immunogenicity scores for the viruses indicated (HERVW, HHV2, HPV). See Table 4 for detailed statistics.

**Figure 9:**
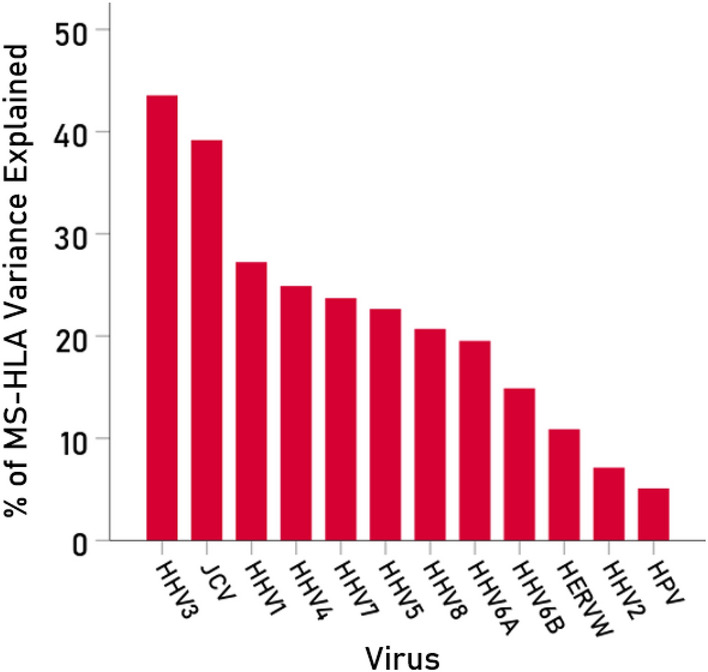
Percent of MS-HLA susceptibility variance explained by V-HLA immunogenicity of the 12 viruses investigated.

Overall, MS-HLA susceptibility scores and V-HLA immunogenicity scores were negatively associated, such that MS-HLA susceptibility scores decreased as the V-HLA immunogenicity increased (Fig. 4; r = − 0.512, P = 0.035, N = 17), indicating a protective effect of viral immunogenicity. In order to evaluate the association of MS-HLA scores with V-HLA immunogenicity of individual viruses in a robust, uniform and nonparametric way, correlations were computed between data converted to normal scores using Blom’s formula^17^. The results are shown in Figs. 5, 6, 7 and 8 as scatterplots of the normalized MS-HLA susceptibility scores vs. normalized V-HLA immunogenicity scores for each of the 12 viruses investigated. It can be seen that all associations were negative, such that MS-HLA susceptibility decreased as V-HLA immunogenicity increased, indicating a protective effect of the latter. The strength of this association differed across viruses (Fig. 9), as reflected in the order of the figures, with Fig. 5 illustrating the case with the strongest association (HHV3), Fig. 8 the case with the weakest association (HPV), and the rest (Figs. 6, 7) in between. Detailed association statistics are given in Table 4, where the strength of MS-HLA susceptibility vs. V-HLA immunogenicity is formalized as the percent of variance in MS-HLA susceptibility scores explained by the corresponding (to each allele) V-HLA immunogenicity. It can be seen (Table 4, Fig. 9) that HHV3 had the highest PVE (43.56%) and HPV the lowest (5.11%), a 8.52 × differential.

**Table 4:** Association statistics between MS-HLA susceptibility scores and immunogenicities of the 12 viruses investigated (N = 17 HLA Class I alleles, Table 1).

Rank	Virus	$r$	SE	Lower 95% CI	Upper 95% CI	P value (1-tailed)	${r}^{2}$	PVE (%)
1	HHV3	− 0.660	0.004	− 0.866	− 0.262	0.002	0.436	43.56
2	JCV	− 0.626	0.007	− 0.851	− 0.208	0.004	0.392	39.19
3	HHV1	− 0.522	0.031	− 0.802	− 0.056	0.016	0.272	27.25
4	HHV4	− 0.499	0.041	− 0.790	− 0.025	0.021	0.249	24.90
5	HHV7	− 0.487	0.047	− 0.784	− 0.008	0.024	0.237	23.72
6	HHV5	− 0.476	0.054	− 0.778	0.006	0.027	0.227	22.66
7	HHV8	− 0.455	0.067	− 0.767	0.033	0.033	0.207	20.70
8	HHV6A	− 0.442	0.075	− 0.761	0.048	0.038	0.195	19.54
9	HHV6B	− 0.386	0.126	− 0.731	0.116	0.063	0.149	14.90
10	HERVW	− 0.330	0.195	− 0.700	0.179	0.195	0.098	10.89
11	HHV2	− 0.267	0.300	− 0.663	0.245	0.300	0.071	7.13
12	HPV	− 0.226	0.384	− 0.637	0.286	0.384	0.192	5.11

$r$ Pearson correlation, *SE* standard error of *r*, *CI* confidence interval, *PVE* percent of the MS-HLA susceptibility scores variance explained by virus immunogenicity. See text for details.

## Discussion

It is largely accepted that MS is a result of complex genetic and environmental interactions. Here we focused on the role of viruses and HLA in MS. Specifically, we evaluated the association between immunogenicity of 12 viruses with respect to 17 HLA Class I alleles that we found to be associated with susceptibility to MS by analyzing population-level epidemiological data. Our findings documented a negative association between the viral V-HLA immunogenicity of all 12 viruses and MS-HLA susceptibility across the 17 MS-HLA Class I susceptibility alleles above. Although the strength of this association varied across viruses, the systematic negative association between viral V-HLA immunogenicity and MS-HLA susceptibility highlight a key role of HLA-mediated virus elimination and/or suppression in influencing MS risk, both at the initial infection and at later relapses caused by reactivation of a latent virus.

MS is presumed to result from exposure to ubiquitous infectious agents in the context of permissive genetic traits^18^. In addition to Class II HLA alleles that have long been implicated in MS^11^, the present findings suggest that the interaction between several common viruses including human herpes viruses and JCV with Class I HLA influences MS prevalence. In light of the role of HLA in antigen elimination and virus suppression, the effect of exposure to certain viruses on MS appears to be moderated by a given HLA allele’s ability to bind and eliminate viral antigens that may otherwise contribute to MS or other conditions. Indeed, HHVs have been implicated in a number of human diseases including MS^4–7^. Following initial infection, typically in childhood, HHVs establish latency and may be periodically reactivated by various triggers and/or waning immunity. Notably, patterns of reactivation have been shown to correspond to MS relapse^19,20^. Similarly, JCV persists in a latent state in the brain, is detectable in human brain tissue, and has also been linked to MS^7,21,22^.The mechanisms underlying the influence of HLA on virus-MS associations are unclear, although several mechanisms including molecular mimicry, persistent viral antigens, bystander activation, superantigen activation, adjuvant effects, epitope spreading, and viral support of autoreactive cell survival have been proposed to explain how viruses might induce autoimmunity in MS^17,23–26^. We have suggested that exposure to pathogens in the absence of HLA that can bind and eliminate those antigens results in antigen persistence and deleterious long-term effects including low-grade chronic inflammation and downstream autoimmunity, apoptosis, and atrophy, thereby setting the groundwork for various conditions including MS^16,27^.

With regard to specific viruses, the strongest effects observed here were for HHV3/VZV, JCV, HHV1/HSV1, HHV4/EBV, HHV7, and HHV5/CMV. Each of these viruses have been previously linked with MS although the findings have been somewhat inconsistent, even for EBV which is considered the leading viral candidate for MS^7,18,21,25,28–38^. For instance, recent evidence demonstrated that although EBV antibodies were higher in MS patients than in controls, neither EBV antibodies nor salivary EBV DNA load were associated with radiological or clinical disease activity in patients with MS^39^. Like many HHVs, EBV is also commonly detected in the healthy adult population^40^ suggesting infection with EBV or other HHVs is insufficient to cause MS in the absence of other factors, including HLA^41,42^. Furthermore, even among HLA alleles that were positively associated with MS risk in the present study, there was considerable variability in HLA-virus immunogencities, MS-HLA susceptibility scores, and their associations.

### Additional contributions

In addition to the contributions of the Class I HLA-virus immunogenicities on MS susceptibility documented here, there are likely other contributing factors. Class II HLA has been strongly linked to MS risk^10^; thus, it is likely that HLA Class II alleles, which are involved in formation of antibodies and immunological memory and often form haplotypes with other HLA alleles including those of Class I, contribute to MS and particularly to autoimmunity associated with MS^26^. Beyond viruses, several other environmental and lifestyle factors also appear to play a role in MS susceptibility including geography, smoking, sun exposure/vitamin D, and adolescent obesity^43–45^. Notably, some of these factors have been shown to interact with HLA to influence MS risk^43^. For example, smoking has been shown to increase the odds of MS in individuals lacking the protective HLA-A*02:01 allele or in carriers of the high-risk Class II HLA-DRB1*15:01 allele^46^. Similar interactions have been documented for obesity^47^. Thus, other HLA x environmental/lifestyle factor interactions not evaluated here may account for some of the unexplained variance in the HLA-MS profile.

### Limitations

Our findings provide novel insights highlighting the interaction of viral exposure and host immunogenetics on MS; however, there are several study limitations that must be considered. First, the analyses here are based on MS diagnosis without regard to subtype; as such, it is unclear to what extent the present findings apply to different forms of the disease. Second, the data utilized here was derived from populations of Continental Western European countries and may not extend to other geographic locations given the global variation in HLA^48,49^, MS prevalence^2^, and virus-MS associations^50,51^. Third, it would be informative to evaluate immunogenicity of these viruses with regard to Class II HLA, particularly in light of the extensive literature documenting the relevance of Class II HLA in MS; however, we are not aware of any in silico application that allows for examination of both binding affinity and immunogenicity for Class II alleles akin to the approach we used here for Class I. Finally, we exclusively focused on the role of viruses in MS and on specific viral proteins, from several possible. The interplay between various environmental factors that have been linked to MS^43–45^ and the HLA-related MS-viral associations remains to be investigated.

## Materials and methods

### Prevalence of MS

The population prevalence of MS was computed for each of 14 countries in Continental Western Europe (Table 5). For each country, we identified the total number of people with each condition in 2019 from the Global Health Data Exchange^52^, a publicly available catalog of data from the Global Burden of Disease study, divided those values by the total population of each country in 2019^52^, and expressed the prevalence as percentage.

**Table 5:** Prevalence of multiple sclerosis in 14 CWE countries in 2019.

	Country	MS prevalence (%)
1	Austria	0.1060
2	Belgium	0.0968
3	Denmark	0.1513
4	Finland	0.1081
5	France	0.1164
6	Germany	0.1194
7	Greece	0.0463
8	Italy	0.1218
9	Netherlands	0.1182
10	Norway	0.1830
11	Portugal	0.0439
12	Spain	0.1090
13	Sweden	0.2051
14	Switzerland	0.1253

### HLA alleles

We obtained the population frequency in 2019 of 69 common HLA Class I alleles from 14 Continental Western European Countries (Austria, Belgium, Denmark, Finland, France, Germany, Greece, Italy, Netherlands, Portugal, Norway, Spain, Sweden, and Switzerland)^53^. The alleles and their mean frequencies (across countries) are given in Table 6.

**Table 6:** The 69 HLA Class I alleles used and their mean frequencies.

Class I								
Gene A			Gene B			Gene C		
	Allele	Frequency		Allele	Frequency		Allele	Frequency
1	A*01:01	0.1170	1	B*07:02	0.1009	1	C*01:02	0.0370
2	A*02:01	0.2715	2	B*08:01	0.0791	2	C*03:03	0.0506
3	A*02:05	0.0122	3	B*13:02	0.0238	3	C*04:01	0.1349
4	A*03:01	0.1501	4	B*14:01	0.0091	4	C*05:01	0.0716
5	A*11:01	0.0527	5	B*14:02	0.0275	5	C*06:02	0.0829
6	A*23:01	0.0237	6	B*15:01	0.0544	6	C*07:01	0.1472
7	A*24:02	0.1051	7	B*15:17	0.0104	7	C*07:02	0.1020
8	A*25:01	0.0139	8	B*15:18	0.0043	8	C*07:04	0.0146
9	A*26:01	0.0356	9	B*18:01	0.0609	9	C*12:02	0.0160
10	A*29:01	0.0058	10	B*27:02	0.0070	10	C*12:03	0.0678
11	A*29:02	0.0315	11	B*27:05	0.0435	11	C*14:02	0.0231
12	A*30:01	0.0165	12	B*35:01	0.0690	12	C*15:02	0.0370
13	A*30:02	0.0132	13	B*35:02	0.0187	13	C*16:01	0.0303
14	A*31:01	0.0295	14	B*35:03	0.0261			
15	A*32:01	0.0368	15	B*35:08	0.0111			
16	A*33:01	0.0116	16	B*37:01	0.0136			
17	A*33:03	0.0066	17	B*38:01	0.0276			
18	A*36:01	0.0063	18	B*39:01	0.0146			
19	A*68:01	0.0353	19	B*39:06	0.0069			
20	A*68:02	0.0220	20	B*40:01	0.0474			
			21	B*40:02	0.0212			
			22	B*41:01	0.0087			
			23	B*41:02	0.0056			
			24	B*44:02	0.0623			
			25	B*44:03	0.0431			
			26	B*44:05	0.0054			
			27	B*45:01	0.0090			
			28	B*47:01	0.0043			
			29	B*49:01	0.0220			
			30	B*50:01	0.0164			
			31	B*51:01	0.0640			
			32	B*52:01	0.0158			
			33	B*55:01	0.0129			
			34	B*56:01	0.0075			
			35	B*57:01	0.0278			
			36	B*58:01	0.0141			

### MS-HLA susceptibility scores

We computed the covariance between the prevalence of MS and the population frequency of the 69 HLA Class I alleles of Table 6:

1 $$\mathrm{MS-HLA \, susceptibility \, score }= \frac{1}{N-1}\sum_{i}^{i=1,N}({f}_{i}-\overline{f })({p}_{i}-\overline{p })$$

where ${f}_{i}, {p}_{i}$ denote allele frequency and MS prevalence for the *i*th country, respectively, and $\overline{f },\overline{p }$ are their means. A positive covariance indicates a positive association between MS prevalence and allele frequency, indicating MS susceptibility.

### Viral antigens

For a given allele, we estimated the immunogenicity of typical proteins of 12 viruses that have been implicated in MS to varying degrees, namely 9 human herpes virus species (HHV1-HHV8), human polyoma JC virus (JCV), human endogenous retrovirus (HERV-W), and human papilloma virus (HPV), the latter of which has not been implicated in MS, to our knowledge, and serves as a negative control, Details of the proteins analyzed are given in Table 7 and their amino acid (AA) sequences are given in the Appendix, together with a short description of their function.

**Table 7:** Viral proteins used.

Virus	Protein description	UniprotKB ID	N (AA)
HHV1	Envelope glycoprotein D	Q69091	394
HHV2	Envelope glycoprotein D	P03172	393
HHV3	Envelope glycoprotein E	Q9J3M8	623
HHV4	Envelope glycoprotein B	P03188	857
HHV5	Envelope glycoprotein B	P06473	906
HHV6A	Glycoprotein Q2	P0DOE0	214
HHV6B	Glycoprotein Q1	Q9QJ11	516
HHV7	Envelope glycoprotein H	P52353	690
HHV8	Envelope glycoprotein H	F5HAK9	730
JCV	Major capsid protein VP1	P03089	354
HPV	Major capsid protein L1	Q81007	494
HERV-W	Envelope protein	Q9UQF0	538

*HHV* human herpes virus, *JCV* human polyomavirus, *HPV* human papillomavirus, *HERV* human endogenous retrovirus.

### Determination of immunogenicity of HLA Class I alleles

**Figure 10:**
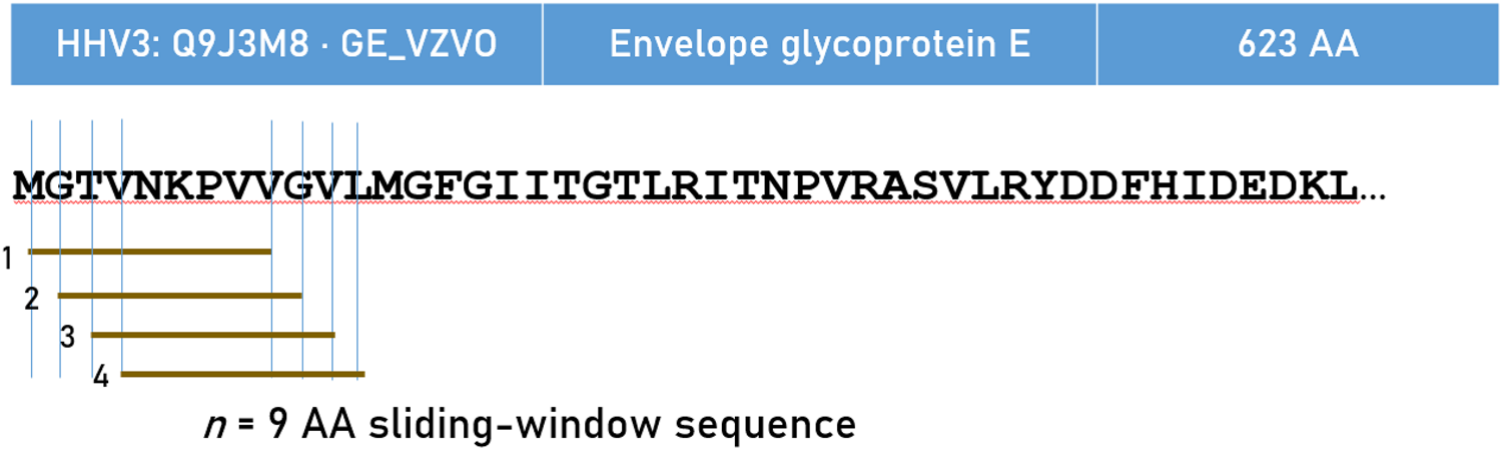
The sliding nonamer window approach used to determine exhaustively in silico the immunogenicity of all possible consecutive nonamers in a protein, illustrated here for HHV3.

The INeo-Epp method^54^ was used for T-cell receptor (TCR) epitope prediction using the INeo-Epp web tool via the INeo-Epp web form interface^55^. For that purpose, we split a given viral antigen (Table 6) to all possible 9-mer (nonamer) AA residue epitopes using a sliding window approach^56–58^ (Fig. 10) and submitted each epitope to the web-application together with a specific HLA allele. More specifically, we paired all epitopes with all alleles and obtained for each pair its percentile rank, a measure of binding affinity of the epitope-HLA allele complex; smaller percentile ranks indicate higher binding affinity. The web-application gave as an outcome a TCR predictive score for pairs with high binding affinities (percentile rank < 2); scores > 0.4 indicated positive immunogenicity and were analyzed further. We computed the following as a comprehensive measure of immunogenicity for quantitative analyses. Let K be the number of nonamers that showed positive immunogenicity (score > 0.4); then, K weighted by their average score $\overline{w }$, would serve as a good estimate of the overall effectiveness of a given allele, *I,* to induce immunogenicity for a given protein:

2 $$\mathrm{V-HLA \, immunogenicity \, score}=\overline{w }K$$

### Association of V-HLA immunogenicities with MS-HLA susceptibility scores

We evaluated the association between MS-HLA susceptibility scores Eq. (1) and V-HLA immunogenicity scores Eq. (2) by computing the Pearson correlation between them for each HLA allele. The correlation coefficient obtained for each virus was squared and multiplied × 100 to provide the percent of MS-HLA susceptibility explained (PVE) by the viral protein immunogenicity:

3 $$PVE=100{r}^{2}$$

### Implementation of analysis procedures

The IBM-SPSS statistical package (version 27) was used for implementing standard statistical analyses, including descriptive statistics and measures of associations. Since we were testing explicitly only a negative association between virus immunogenicity and MS-HLA covariance, one-sided P-values were used. We did not correct for multiple comparisons because these were planned comparisons.

### Ethical approval

This article does not contain any studies with human participants performed by any of the authors.

### Supplementary Information

Supplementary Information.

## Data Availability

All data used were retrieved from freely accessible websites [^52^; http://ghdx.healthdata.org/gbd-results-tool], [^53^; http://allelefrequencies.net/hla6006a.asp], and, as such, are publicly and freely available.

## References

[1] Dobson R, Giovannoni G (2019). Multiple sclerosis: A review. Eur. J. Neurol..

[2] GBD 2016 Multiple Sclerosis Collaborators (2019). Global, regional, and national burden of multiple sclerosis 1990–2016: A systematic analysis for the Global Burden of Disease Study 2016. Lancet Neurol..

[3] Walton C (2020). Rising prevalence of multiple sclerosis worldwide: Insights from the Atlas of MS, third edition. Mult. Scler..

[4] Virtanen JO, Jacobson S (2012). Viruses and multiple sclerosis. CNS Neurol. Disord. Drug Targets..

[5] Pormohammad A, Azimi T, Falah F, Faghihloo E (2018). Relationship of human herpes virus 6 and multiple sclerosis: A systematic review and meta-analysis. J. Cell Physiol..

[6] Donati D (2020). Viral infections and multiple sclerosis. Drug Discov. Today Dis. Models..

[7] Tarlinton RE, Martynova E, Rizvanov AA, Khaiboullina S, Verma S (2020). Role of viruses in the pathogenesis of multiple sclerosis. Viruses..

[8] Stoner GL (1993). Polyomavirus models of brain infection and the pathogenesis of multiple sclerosis. Brain Pathol..

[9] Outteryck O (2014). JC-virus seroconversion in multiple sclerosis patients receiving natalizumab. Mult. Scler. J..

[10] Ho PR (2017). Risk of natalizumab-associated progressive multifocal leukoencephalopathy in patients with multiple sclerosis: A retrospective analysis of data from four clinical studies. Lancet Neurol..

[11] Hollenbach JA, Oksenberg JR (2015). The immunogenetics of multiple sclerosis: A comprehensive review. J. Autoimmun..

[12] Sawcer S (2011). Genetic risk and a primary role for cell-mediated immune mechanisms in multiple sclerosis. Nature..

[13] Sawcer S, Franklin RJ, Ban M (2014). Multiple sclerosis genetics. Lancet Neurol..

[14] Baranzini SE, Oksenberg JR (2017). The genetics of multiple sclerosis: from 0 to 200 in 50 years. Trends Genet..

[15] International Multiple Sclerosis Genetics Consortium (2019). Multiple sclerosis genomic map implicates peripheral immune cells and microglia in susceptibility. Science.

[16] James LM, Georgopoulos AP (2021). Immunogenetic epidemiology of multiple sclerosis in 14 continental western European countries. J. Immunol. Sci..

[17] Blom G (1958). Statistical Estimates and Transformed Beta-Variables.

[18] Kakalacheva K, Münz C, Lünemann JD (2011). Viral triggers of multiple sclerosis. Biochim. Biophys. Acta..

[19] Sotelo J, Ordoñez G, Pineda B, Flores J (2014). The participation of varicella zoster virus in relapses of multiple sclerosis. Clin. Neurol. Neurosurg..

[20] Sotelo J, Martínez-Palomo A, Ordoñez G, Pineda B (2008). Varicella-zoster virus in cerebrospinal fluid at relapses of multiple sclerosis. Ann. Neurol..

[21] Del Valle L (2002). Expression of JC virus T-antigen in a patient with MS and glioblastoma multiforme. Neurology.

[22] White FA, Ishaq M, Stoner GL, Frisque RJ (1992). JC virus DNA is present in many human brain samples from patients without progressive multifocal leukoencephalopathy. J. Virol..

[23] Aloisi F, Giovannoni G, Salvetti M (2023). Epstein-Barr virus as a cause of multiple sclerosis: Opportunities for prevention and therapy. Lancet Neurol..

[24] Fujinami RS, von Herrath MG, Christen U, Whitton JL (2006). Molecular mimicry, bystander activation, or viral persistence: Infections and autoimmune disease. Clin. Microbiol. Rev..

[25] Jakhmola S, Upadhyay A, Jain K, Mishra A, Jha HC (2021). Herpesviruses and the hidden links to multiple sclerosis neuropathology. J. Neuroimmunol..

[26] Gough SC, Simmonds MJ (2007). The HLA region and autoimmune disease: Associations and mechanisms of action. Curr. Genom..

[27] Georgopoulos AP, James LM (2018). Persistent antigens hypothesis: The human leukocyte antigen (HLA) connection. J. Neurol. Neuromed..

[28] Opsahl ML, Kennedy PG (2006). Investigating the presence of human herpesvirus 7 and 8 in multiple sclerosis and normal control brain tissue. J. Neurol. Sci..

[29] Duarte LF (2022). Is there a role for herpes simplex virus type 1 in multiple sclerosis?. Microb. Infect..

[30] Xu L (2021). Positive association of herpes simplex virus-IgG with multiple sclerosis: A systematic review and meta-analysis. Mult. Scler. Relat. Disord..

[31] Manouchehrinia A (2017). Prevalence of a history of prior varicella/herpes zoster infection in multiple sclerosis. J. Neurovirol..

[32] Khalesi Z (2023). Association between human herpesviruses and multiple sclerosis: A systematic review and meta-analysis. Microb. Pathog..

[33] Nora-Krukle Z (2011). Human herpesvirus 6 and 7 reactivation and disease activity in multiple sclerosis. Medicina..

[34] Taus C (2000). Absence of HHV-6 and HHV-7 in cerebrospinal fluid in relapsing-remitting multiple sclerosis. Acta Neurol. Scand..

[35] Vanheusden M, Stinissen P, Hart BA, Hellings N (2015). Cytomegalovirus: A culprit or protector in multiple sclerosis?. Trends Mol. Med..

[36] Ascherio A, Munger KL (2010). Epstein-Barr virus infection and multiple sclerosis: A review. J. Neuroimmune Pharmacol..

[37] Soldan SS, Lieberman PM (2023). Epstein-Barr virus and multiple sclerosis. Nat. Rev. Microbiol..

[38] Bjornevik K (2022). Longitudinal analysis reveals high prevalence of Epstein-Barr virus associated with multiple sclerosis. Science..

[39] Gieß RM (2017). Epstein-Barr virus antibodies in serum and DNA load in saliva are not associated with radiological or clinical disease activity in patients with early multiple sclerosis. PLoS ONE..

[40] Cohen JI (2000). Epstein-Barr virus infection. New Engl. J. Med..

[41] Sundqvist E (2012). Epstein-Barr virus and multiple sclerosis: Interaction with HLA. Genes Immun..

[42] Agostini S (2018). HLA alleles modulate EBV viral load in multiple sclerosis. J. Transl. Med..

[43] Alfredsson L, Olsson T (2019). Lifestyle and environmental factors in multiple sclerosis. Cold Spring Harb. Perspect. Med..

[44] Olsson T, Barcellos L, Alfredsson L (2017). Interactions between genetic, lifestyle and environmental risk factors for multiple sclerosis. Nat. Rev. Neurol..

[45] Ascherio A (2013). Environmental factors in multiple sclerosis. Expert Rev. Neurother..

[46] Hedstrom AK (2011). Smoking and two human leukocyte antigen genes interact at to increase the risk for multiple sclerosis. Brain..

[47] Hedstrom AK (2014). Interaction between adolescent obesity and HLA risk genes in the etiology of multiple sclerosis. Neurology..

[48] Garamszegi LZ (2014). Global distribution of malaria−resistant MHC−HLA alleles: The number and frequencies of alleles and malaria risk. Malar. J..

[49] Singh R (2007). A comparative review of HLA associations with hepatitis B and C viral infections across global populations. World J. Gastroenterol..

[50] Thakolwiboon S (2020). Regional differences in the association of cytomegalovirus seropositivity and multiple sclerosis: A systematic review and meta-analysis. Mult. Scler. Relat. Disord..

[51] Rice EM, Thakolwiboon S, Avila M (2021). Geographic heterogeneity in the association of varicella-zoster virus seropositivity and multiple sclerosis: A systematic review and meta-analysis. Mult. Scler. Relat. Disord..

[52] Global Burden of Disease Collaborative Network. *Global Burden of Disease Study 2019 (GBD 2019) Results*. (Institute for Health Metrics and Evaluation (IHME), 2020). http://ghdx.healthdata.org/gbd-results-tool. Accessed 24 April 2023.

[53] Gonzalez-Galarza FF (2020). Allele frequency net database (AFND): 2020 update: Gold-standard data classification, open access genotype data and new query tools. Nucleic Acid Res..

[54] Wang G (2020). INeo-Epp: A novel T-cell HLA class-I immunogenicity or neoantigenic epitope prediction method based on sequence-related amino acid features. Biomed. Res. Int..

[55] Website: INeo-Epp: A novel T-cell HLA class-I immunogenicity prediction method. http://www.biostatistics.online/ineo-epp/neoantigen.php 10.1155/2020/5798356 PMC7315274 32626747

[56] Charonis S, James LM, Georgopoulos AP (2020). In silico assessment of binding affinities of three dementia-protective human leukocyte antigen (HLA) alleles to nine human herpes virus antigens. Curr. Res. Transl. Med..

[57] Charonis S, Tsilibary EP, Georgopoulos AP (2020). SARS-CoV-2 virus and human leukocyte antigen (HLA) Class II: Investigation in silico of binding affinities for COVID-19 protection and vaccine development. J. Immunol. Sci..

[58] Charonis SA, Tsilibary EP, Georgopoulos AP (2021). In silico investigation of binding affinities between human leukocyte antigen class I molecules and SARS-CoV-2 virus spike and ORF1ab proteins. Explor. Immunol..

