# Supplementary material for: Negative association between multiple sclerosis immunogenetic profile and in silico immunogenicities of 12 viruses
Source: Sci Rep. 2023 Oct 31;13:18654. doi: 10.1038/s41598-023-45931-5 (PMC10618254; doi:10.1038/s41598-023-45931-5)
Supplement: Supplementary file 1 — Supplementary Information. [file 41598_2023_45931_MOESM1_ESM.docx]

**APPENDIX**

Amino acid sequences of the 9 HHV strains (Table 3), human polyoma virus (JCV), human papilloma virus, and human endogenous retrovirus HERV-W. Strain labels are from Uniprot (https://www.uniprot.org/uniprotkb/)

| HHV1: Q69091 · GD_HHV11 | Envelope glycoprotein D | 394 AA |
| --- | --- | --- |

MGGAAARLGAVILFVVIVGLHGVRSKYALVDASLKMADPNRFRGKDLPVLDQLTDPPGVRRVYHIQAGLPDPFQPPSLPITVYYAVLERACRSVLLNAPSEAPQIVRGASEDVRKQPYNLTIAWFRMGGNCAIPITVMEYTECSYNKSLGACPIRTQPRWNYYDSFSAVSEDNLGFLMHAPAFETAGTYLRLVKINDWTEITQFILEHRAKGSCKYALPLRIPPSACLSPQAYQQGVTVDSIGMLPRFIPENQRTVAVYSLKIAGWHGPKAPYTSTLLPPELSETPNATQPELAPEDPEDSALLEDPVGTVAPQIPPNWHIPSIQDAATPYHPPATPNNMGLIAGAVGGSLLAALVICGIVYWMRRHTQKAPKRIRLPHIREDDQPSSHQPLFY

| HHV2: P03172 · GD_HHV23 | Envelope glycoprotein D | 393 AA |
| --- | --- | --- |

MGRLTSGVGTAALLVVAVGLRVVCAKYALADPSLKMADPNRFRGKNLPVLDRLTDPPGVKRVYHIQPSLEDPFQPPSIPITVYYAVLERACRSVLLHAPSEAPQIVRGASDEARKHTYNLTIAWYRMGDNCAIPITVMEYTECPYNKSLGVCPIRTQPRWSYYDSFSAVSEDNLGFLMHAPAFETAGTYLRLVKINDWTEITQFILEHRARASCKYALPLRIPPAACLTSKAYQQGVTVDSIGMLPRFIPENQRTVALYSLKIAGWHGPKPPYTSTLLPPELSDTTNATQPELVPEDPEDSALLEDPAGTVSSQIPPNWHIPSIQDVAPHHAPAAPSNPGLIIGALAGSTLAVLVIGGIAFWVRRRAQMAPKRLRLPHIRDDDAPPSHQPLFY

| HHV3: Q9J3M8 · GE_VZVO | Envelope glycoprotein E | 623 AA |
| --- | --- | --- |

MGTVNKPVVGVLMGFGIITGTLRITNPVRASVLRYDDFHIDEDKLDTNSVYEPYYHSDHAESSWVNRGESSRKAYDHNSPYIWPRNDYDGFLENAHEHHGVYNQGRGIDSGERLMQPTQMSAQEDLGDDTGIHVIPTLNGDDRHKIVNVDQRQYGDVFKGDLNPKPQGQRLIEVSVEENHPFTLRAPIQRIYGVRYTETWSFLPSLTCTGDAAPAIQHICLKHTTCFQDVVVDVDCAENTKEDQLAEISYRFQGKKEADQPWIVVNTSTLFDELELDPPEIEPGVLKVLRTEKQYLGVYIWNMRGSDGTSTYATFLVTWKGDEKTRNPTPAVTPQPRGAEFHMWNYHSHVFSVGDTFSLAMHLQYKIHEAPFDLLLEWLYVPIDPTCQPMRLYSTCLYHPNAPQCLSHMNSGCTFTSPHLAQRVASTVYQNCEHADNYTAYCLGISHMEPSFGLILHDGGTTLKFVDTPESLSGLYVFVVYFNGHVEAVAYTVVSTVDHFVNAIEERGFPPTAGQPPATTKPKEITPVNPGTSPLLRYAAWTGGLAAVVLLCLVIFLICTAKRMRVKAYRVDKSPYNQSMYYAGLPVDDFEDSESTDTEEEFGNAIGGSHGGSSYTVYIDKTR

| HHV4: P03188 · GB_EBVB9 | Envelope glycoprotein B | 857 AA |
| --- | --- | --- |

MTRRRVLSVVVLLAALACRLGAQTPEQPAPPATTVQPTATRQQTSFPFRVCELSSHGDLFRFSSDIQCPSFGTRENHTEGLLMVFKDNIIPYSFKVRSYTKIVTNILIYNGWYADSVTNRHEEKFSVDSYETDQMDTIYQCYNAVKMTKDGLTRVYVDRDGVNITVNLKPTGGLANGVRRYASQTELYDAPGWLIWTYRTRTTVNCLITDMMAKSNSPFDFFVTTTGQTVEMSPFYDGKNKETFHERADSFHVRTNYKIVDYDNRGTNPQGERRAFLDKGTYTLSWKLENRTAYCPLQHWQTFDSTIATETGKSIHFVTDEGTSSFVTNTTVGIELPDAFKCIEEQVNKTMHEKYEAVQDRYTKGQEAITYFITSGGLLLAWLPLTPRSLATVKNLTELTTPTSSPPSSPSPPAPSAARGSTPAAVLRRRRRDAGNATTPVPPTAPGKSLGTLNNPATVQIQFAYDSLRRQINRMLGDLARAWCLEQKRQNMVLRELTKINPTTVMSSIYGKAVAAKRLGDVISVSQCVPVNQATVTLRKSMRVPGSETMCYSRPLVSFSFINDTKTYEGQLGTDNEIFLTKKMTEVCQATSQYYFQSGNEIHVYNDYHHFKTIELDGIATLQTFISLNTSLIENIDFASLELYSRDEQRASNVFDLEGIFREYNFQAQNIAGLRKDLDNAVSNGRNQFVDGLGELMDSLGSVGQSITNLVSTVGGLFSSLVSGFISFFKNPFGGMLILVLVAGVVILVISLTRRTRQMSQQPVQMLYPGIDELAQQHASGEGPGINPISKTELQAIMLALHEQNQEQKRAAQRAAGPSVASRALQAARDRFPGLRRRRYHDPETAAALLGEAETEF

| HHV5: P06473 · GB_HCMVA | Envelope glycoprotein B | gB | 906 AA |
| --- | --- | --- | --- |

MESRIWCLVVCVNLCIVCLGAAVSSSSTSHATSSTHNGSHTSRTTSAQTRSVYSQHVTSSEAVSHRANETIYNTTLKYGDVVGVNTTKYPYRVCSMAQGTDLIRFERNIICTSMKPINEDLDEGIMVVYKRNIVAHTFKVRVYQKVLTFRRSYAYIYTTYLLGSNTEYVAPPMWEIHHINKFAQCYSSYSRVIGGTVFVAYHRDSYENKTMQLIPDDYSNTHSTRYVTVKDQWHSRGSTWLYRETCNLNCMLTITTARSKYPYHFFATSTGDVVYISPFYNGTNRNASYFGENADKFFIFPNYTIVSDFGRPNAAPETHRLVAFLERADSVISWDIQDEKNVTCQLTFWEASERTIRSEAEDSYHFSSAKMTATFLSKKQEVNMSDSALDCVRDEAINKLQQIFNTSYNQTYEKYGNVSVFETSGGLVVFWQGIKQKSLVELERLANRSSLNITHRTRRSTSDNNTTHLSSMESVHNLVYAQLQFTYDTLRGYINRALAQIAEAWCVDQRRTLEVFKELSKINPSAILSAIYNKPIAARFMGDVLGLASCVTINQTSVKVLRDMNVKESPGRCYSRPVVIFNFANSSYVQYGQLGEDNEILLGNHRTEECQLPSLKIFIAGNSAYEYVDYLFKRMIDLSSISTVDSMIALDIDPLENTDFRVLELYSQKELRSSNVFDLEEIMREFNSYKQRVKYVEDKVVDPLPPYLKGLDDLMSGLGAAGKAVGVAIGAVGGAVASVVEGVATFLKNPFGAFTIILVAIAVVIITYLIYTRQRRLCTQPLQNLFPYLVSADGTTVTSGSTKDTSLQAPPSYEESVYNSGRKGPGPPSSDASTAAPPYTNEQAYQMLLALARLDAEQRAQQNGTDSLDGQTGTQDKGQKPNLLDRLRHRKNGYRHLKDSDEEENV

| HHV6A: P0DOE0 · GQ2_HHV6U | Envelope glycoprotein Q2 | 214 AA |
| --- | --- | --- |

MHFLVVYILIHFHAYRGMAALPLFSTLPKITSCCDSYVVINSSTSVSSLISTCLDGEILFQNEGQKFCRPLTDNRTIVYTMQDQVQKPLSVTWMDFNLVISDYGRDVINNLTKSAMLARKNGPRYLQMENGPRYLQMETRISDLFRHECYQDNYYVLDKKLQMFYPTTHSNELLFYPSEATLPSPWQEPPFSSPWPEPTFPSRWYWLLLNYTNY

| HHV6B: Q9QJ11 · GQ1_HHV6Z | Envelope glycoprotein Q1 | 516 AA |
| --- | --- | --- |

MRPPRRSAPILVCAISMATALSNATVHRDAGTVESTPPPDDEDNYTAKYYDDSIYFNIYDGTNPTPRRRTLPEIISKFSTSEMSRLGGLKAFVPVDYTPTTTLEDIEDLLNYAICDDNSCGCLIETEARXMFGDIIICVPLSAESRGVRNLKSRIMPMGLSQILSSGLGLHFSLLYGAFGSNYNSLAYMERLKPLTAMTAIAFCPMTSKLELRQNYRLEKARXNLIVNIELLKIQNHGGQTIKTLTSFAIVRKDSDGQDWETCTRFASVSIEDILRSKPAANGTCCPPRDVHHDRPTLQSSNSWTRTEYFEPWQDVVDAYVPINDNHCPNDSYVVFQTLQGHEWCSRLNKNDTKNYLSSVLAFKNALYETEELMETIGMRLASQILSLVGQRGTSIRNIDPAIVSALWHSLPEKLTTTNIKYDIASPTHMSPALXTIFIQTGTSKQRFRNAGLLMVNNIFTVQARYSKQNMFEKKIYGYEHLGQALCEGGHVFYNPRDVYFQNIKMAATEPTVVRT

| HHV7: P52353 · GH_HHV7J | Envelope glycoprotein H | 690 AA |
| --- | --- | --- |

MYFYINSLLLIVSINGWKHWNILNSSICVNEKTNQTIIQPGLITFNFHDYNETRVYQIPKCLFGYTFVSNLFDSVNFDESFDQYKHRITRFFNPSTEKAVKIYAQKFQTNIKPVSHTKTITVSFLPLFYEKDVYFANVSEIRKLYYNQYICTLSNGLTDYLFPITERCVMRHYNYLNTVFMLALTPSFFIISVETGMDDVVFIFGNVSRIFFKAPFRKSSFIYRQTVSDDLLLITKKTTIERFYPFLKIDFLDDIWKQNYDISFLIAKFNKLATVYIMEGFCGKPVNKDTFHLMFLFGLTHFLYSTRGDGLLPLLEILNTHQSIITMGRFLEKCFKMTKSHLLYPEMEKLQNFQLVDYSYITSDLTIPISAKLAFLSLADGRIVTVPQNKWKEIENNIETLYEKHKLFTNLTQPERANLFLLSEIGNSLVFQEKIKRKIHVLLASLCNPLEMYFWTHMLDNVMDIETMFSPCATATRKDLTQRVVNNILSYKNLDAYTNKVMNTLSVYRKKRLDMFKSISCVSNEQAAFLTLPNITYTISSKYILAGTSFSVTSTVISTTIIITVVPLNSTCTPTNYKYSVKNIKPIYNISSHDCVFCESLVVEYDDIDGIIQFVYIMDDKQLLKLIDPDTNFIDVNPRTHYLLFLRNGSVFEITALDLKSSQVSIMLVLLYLIIIIIVLFGIYHVFRLF

| HHV8: F5HAK9 · GH_HHV8P | Envelope glycoprotein H | 730 AA |
| --- | --- | --- |

MQGLAFLAALACWRCISLTCGATGALPTTATTITRSATQLINGRTNLSIELEFNGTSFFLNWQNLLNVITEPALTELWTSAEVAEDLRVTLKKRQSLFFPNKTVVISGDGHRYTCEVPTSSQTYNITKGFNYSALPGHLGGFGINARLVLGDIFASKWSLFARDTPEYRVFYPMNVMAVKFSISIGNNESGVALYGVVSEDFVVVTLHNRSKEANETASHLLFGLPDSLPSLKGHATYDELTFARNAKYALVAILPKDSYQTLLTENYTRIFLNMTESTPLEFTRTIQTRIVSIEARRACAAQEAAPDIFLVLFQMLVAHFLVARGIAEHRFVEVDCVCRQYAELYFLRRISRLCMPTFTTVGYNHTTLGAVAATQIARVSATKLASLPRSSQETVLAMVQLGARDGAVPSSILEGIAMVVEHMYTAYTYVYTLGDTERKLMLDIHTVLTDSCPPKDSGVSEKLLRTYLMFTSMCTNIELGEMIARFSKPDSLNIYRAFSPCFLGLRYDLHPAKLRAEAPQSSALTRTAVARGTSGFAELLHALHLDSLNLIPAINCSKITADKIIATVPLPHVTYIISSEALSNAVVYEVSEIFLKSAMFISAIKPDCSGFNFSQIDRHIPIVYNISTPRRGCPLCDSVIMSYDESDGLQSLMYVTNERVQTNLFLDKSPFFDNNNLHIHYLWLRDNGTVVEIRGMYRRRAASALFLILSFIGFSGVIYFLYRLFSILY

| JC polyomavirus (JCV)  P03089 VP1_POVJC | Major capsid protein VP1 | 354 AA |
| --- | --- | --- |

MAPTKRKGERKDPVQVPKLLIRGGVEVLEVKTGVDSITEVECFLTPEMGDPDEHLRGFSKSISISDTFESDSPNRDMLPCYSVARIPLPNLNEDLTCGNILMWEAVTLKTEVIGVTSLMNVHSNGQATHDNGAGKPVQGTSFHFFSVGGEALELQGVLFNYRTKYPDGTIFPKNATVQSQVMNTEHKAYLDKNKAYPVECWVPDPTRNENTRYFGTLTGGENVPPVLHITNTATTVLLDEFGVGPLCKGDNLYLSAVDVCGMFTNRSGSQQWRGLSRYFKVQLRKRRVKNPYPISFLLTDLINRRTPRVDGQPMYGMDAQVEEVRVFEGTEELPGDPDMMRYVDKYGQLQTKML

| Human papillomavirus Q81007 | Major capsid protein L1 | 494 AA |
| --- | --- | --- |

TVYLPPVPVSKVVSTDEYVARTNIYYHAGTSRLLAVGHPYFPIKKPNNNKILVPKVSGLQYRVFRIHLPDPNKFGFPDTSFYNPDTQRLVWACVGVEVGRGQPLGVGISGHPLLNKLDDTENASAYAANAGVDNRECISMDYKQTQLCLIGCKPPIGEHWGKGSPCTNVAVNPGDCPPLELINTVIQDGDMVDTGFGAMDFTTLQANKSEVPLDICTSICKYPDYIKMVSEPYGDSLFFYLRREQMFVRHLFNRAGTVGENVPDDLYIKGSGSTANLASSNYFPTPSGSMVTSDAQIFNKPYWLQRAQGHNNGICWGNQLFVTVVDTTRSTNMSLCAAISTSETTYKNTNFKEYLRHGEEYDLQFIFQLCKITLTADVMTYIHSMNSTILEDWNFGLQPPPGGTLEDTYRFVTSQAIACQKHTPPAPKEDPLKKYTFWEVNLKEKFSADLDQFPLGRKFLLQAGLKAKPKFTLGKRKATPTTSSTSTTAKRKKR

| Human Endogenous Retrovirus HERV W Q9UQF0 | Envelope protein | 538 AA |
| --- | --- | --- |

MALPYHIFLFTVLLPSFTLTAPPPCRCMTSSSPYQEFLWRMQRPGNIDAPSYRSLSKGTPTFTAHTHMPRNCYHSATLCMHANTHYWTGKMINPSCPGGLGVTVCWTYFTQTGMSDGGGVQDQAREKHVKEVISQLTRVHGTSSPYKGLDLSKLHETLRTHTRLVSLFNTTLTGLHEVSAQNPTNCWICLPLNFRPYVSIPVPEQWNNFSTEINTTSVLVGPLVSNLEITHTSNLTCVKFSNTTYTTNSQCIRWVTPPTQIVCLPSGIFFVCGTSAYRCLNGSSESMCFLSFLVPPMTIYTEQDLYSYVISKPRNKRVPILPFVIGAGVLGALGTGIGGITTSTQFYYKLSQELNGDMERVADSLVTLQDQLNSLAAVVLQNRRALDLLTAERGGTCLFLGEECCYYVNQSGIVTEKVKEIRDRIQRRAEELRNTGPWGLLSQWMPWILPFLGPLAAIILLLLFGPCIFNLLVNFVSSRIEAVKLQMEPKMQSKTKIYRRPLDRPASPRSDVNDIKGTPPEEISAAQPLLRPNSAGSS

**Functions of proteins above (copied from the UNIPROT site mentioned; accessed on October 2, 2023)**

**HHV1** Envelope glycoprotein that binds to the potential host cell entry receptors TNFRSF14/HVEM and NECTIN1. (https://www.uniprot.org/uniprotkb/Q69091/entry)

**HHV2** Envelope glycoprotein that binds to the potential host cell entry receptors TNFRSF14/HVEM and NECTIN1. (https://www.uniprot.org/uniprotkb/P03172/entry)

**HHV3** Envelope glycoprotein that binds to the potential host cell entry receptor IDE. (https://www.uniprot.org/uniprotkb/ Q9J3M8/entry)

**HHV4** Envelope glycoprotein that forms spikes at the surface of virion envelope. Essential for the initial attachment to heparan sulfate moieties of the host cell surface proteoglycans. Involved in fusion of viral and cellular membranes leading to virus entry into the host cell. Following initial binding to its host receptors, membrane fusion is mediated by the fusion machinery composed at least of gB and the heterodimer gH/gL. May be involved in the fusion between the virion envelope and the outer nuclear membrane during virion egress. (https://www.uniprot.org/uniprotkb/ P03188/entry)

**HHV5** Envelope glycoprotein that plays a role in host cell entry, cell to-cell virus transmission, and fusion of infected cells. May be involved in the initial attachment via binding to heparan sulfate together with the gM/gN complex that binds heparin with higher affinity. Interacts with host integrin ITGB1, PDGFRA and EGFR that likely serve as postattachment entry receptors. Participates also in the fusion of viral and cellular membranes leading to virus entry into the host cell. Membrane fusion is mediated by the fusion machinery composed at least of gB and the heterodimer gH/gL (https://www.uniprot.org/uniprotkb/P06473/entry)

**HHV6A** Plays a role in virus entry by participating in host receptor binding at the cell surface. ([https://www.uniprot.org/uniprotkb/ P0DOE0/entry](https://www.uniprot.org/uniprotkb/%20P0DOE0/entry))

**HHV6B** Plays a role in virus entry by participating in host receptor binding at the cell surface. (<https://www.uniprot.org/uniprotkb/Q9QJ11/entry>)

**HHV7** The heterodimer glycoprotein H-glycoprotein L is required for the fusion of viral and plasma membranes leading to virus entry into the host cell. Following initial binding to host receptor, membrane fusion is mediated by the fusion machinery composed of gB and the heterodimer gH/gL. May also be involved in the fusion between the virion envelope and the outer nuclear membrane during virion morphogenesis. (https://www.uniprot.org/uniprotkb/P52353/entry)

**HHV8** The heterodimer glycoprotein H-glycoprotein L is required for the fusion of viral and plasma membranes leading to virus entry into the host cell. Following initial binding to host receptor, membrane fusion is mediated by the fusion machinery composed of gB and the heterodimer gH/gL. May also be involved in the fusion between the virion envelope and the outer nuclear membrane during virion morphogenesis (By similarity).
Targets host EPHA2 to promote KSHV entry. (<https://www.uniprot.org/uniprotkb/F5HAK9/entry>)

**JCV** Forms an icosahedral capsid with a T=7 symmetry and a 40 nm diameter. The capsid is composed of 72 pentamers linked to each other by disulfide bonds and associated with VP2 or VP3 proteins. Interacts with a N-linked glycoprotein containing terminal alpha(2-6)-linked sialic acids on the cell surface to provide virion attachment to target cell. The serotonergic receptor 5HT2AR also acts as a cellular receptor for JCV on human glial cells. Once attached, the virions enter predominantly by a ligand-inducible clathrin-dependent pathway and traffic to the ER. Inside the endoplasmic reticulum, the protein folding machinery isomerizes VP1 interpentamer disulfide bonds, thereby triggering initial uncoating. Next, the virion uses the endoplasmic reticulum-associated degradation machinery to probably translocate in the cytosol before reaching the nucleus. Nuclear entry of the viral DNA involves the selective exposure and importin recognition of VP2/Vp3 nuclear localization signal. In late phase of infection, neo-synthesized VP1 encapsulates replicated genomic DNA at nuclear domains called promyelocytic leukemia (PML) bodies, and participates in rearranging nucleosomes around the viral DNA. ([https://www.uniprot.org/uniprotkb/ P03089/entry](https://www.uniprot.org/uniprotkb/%20P03089/entry))

**HPV** Forms an icosahedral capsid with a T=7 symmetry and a 50 nm diameter. The capsid is composed of 72 pentamers linked to each other by disulfide bonds and associated with L2 proteins. Binds to heparan sulfate proteoglycans on cell surface of basal layer keratinocytes to provide initial virion attachment. This binding mediates a conformational change in the virus capsid that facilitates efficient infection. The virion enters the host cell via endocytosis. During virus trafficking, L1 protein dissociates from the viral DNA and the genomic DNA is released to the host nucleus. The virion assembly takes place within the cell nucleus. Encapsulates the genomic DNA together with protein L2. ([https://www.uniprot.org/uniprotkb/ Q81007/entry](https://www.uniprot.org/uniprotkb/%20Q81007/entry))

**HERV-W** This endogenous retroviral envelope protein has retained its original fusogenic properties and participates in trophoblast fusion and the formation of a syncytium during placenta morphogenesis. May induce fusion through binding of SLC1A4 and SLC1A5. ([https://www.uniprot.org/uniprotkb/ Q9UQF0/entry](https://www.uniprot.org/uniprotkb/%20Q9UQF0/entry))
